# Federated causal inference based on real-world observational data sources: application to a SARS-CoV-2 vaccine effectiveness assessment

**DOI:** 10.1186/s12874-023-02068-3

**Published:** 2023-10-23

**Authors:** Marjan Meurisse, Francisco Estupiñán-Romero, Javier González-Galindo, Natalia Martínez-Lizaga, Santiago Royo-Sierra, Simon Saldner, Lorenz Dolanski-Aghamanoukjan, Alexander Degelsegger-Marquez, Stian Soiland-Reyes, Nina Van Goethem, Enrique Bernal-Delgado, for the BeYond-COVID project

**Affiliations:** 1 Department of Epidemiology and Public Health, Sciensano, Brussels, Belgium; 2 IREC – EPID, Université Catholique de Louvain, Brussels, Belgium; 3 Data science for Health Services and Policy, Instituto Aragonés de Ciencias de la Salud (IACS), Zaragoza, Spain; 4 Data Archiving and Networked Services, Royal Netherlands Academy of Arts & Sciences, Amsterdam, The Netherlands; 5 International Affairs, Policy, Evaluation and Digitalisation, Gesundheit Österreich GmbH (GÖG), Vienna, Austria; 6 Department of Computer Science, The University of Manchester, Manchester, UK; 7 Informatics Institute, Universiteit van Amsterdam, Amsterdam, The Netherlands

**Keywords:** Federated analysis, Causal inference, Real-world data, Comparative effectiveness, Vaccines, COVID-19, Pandemic preparedness

## Abstract

**Introduction:**

Causal inference helps researchers and policy-makers to evaluate public health interventions. When comparing interventions or public health programs by leveraging observational sensitive individual-level data from populations crossing jurisdictional borders, a federated approach (as opposed to a pooling data approach) can be used. Approaching causal inference by re-using routinely collected observational data across different regions in a federated manner, is challenging and guidance is currently lacking. With the aim of filling this gap and allowing a rapid response in the case of a next pandemic, a methodological framework to develop studies attempting causal inference using federated cross-national sensitive observational data, is described and showcased within the European BeYond-COVID project.

**Methods:**

A framework for approaching federated causal inference by re-using routinely collected observational data across different regions, based on principles of legal, organizational, semantic and technical interoperability, is proposed. The framework includes step-by-step guidance, from defining a research question, to establishing a causal model, identifying and specifying data requirements in a common data model, generating synthetic data, and developing an interoperable and reproducible analytical pipeline for distributed deployment. The conceptual and instrumental phase of the framework was demonstrated and an analytical pipeline implementing federated causal inference was prototyped using open-source software in preparation for the assessment of real-world effectiveness of SARS-CoV-2 primary vaccination in preventing infection in populations spanning different countries, integrating a data quality assessment, imputation of missing values, matching of exposed to unexposed individuals based on confounders identified in the causal model and a survival analysis within the matched population.

**Results:**

The conceptual and instrumental phase of the proposed methodological framework was successfully demonstrated within the BY-COVID project. Different Findable, Accessible, Interoperable and Reusable (FAIR) research objects were produced, such as a study protocol, a data management plan, a common data model, a synthetic dataset and an interoperable analytical pipeline.

**Conclusions:**

The framework provides a systematic approach to address federated cross-national policy-relevant causal research questions based on sensitive population, health and care data in a privacy-preserving and interoperable way. The methodology and derived research objects can be re-used and contribute to pandemic preparedness.

**Supplementary Information:**

The online version contains supplementary material available at 10.1186/s12874-023-02068-3.

## Background

Causal inference, the process of estimating a causal effect of interest (e.g., of a treatment or intervention on a health outcome), is a major interest in public health research. Identifying causal relationships can signal targets for public health policy (e.g., increase exposure to a beneficial determinant or treatment, or reduce exposure to a hazardous one) or allows the evaluation of public health interventions. Estimating causal effects for public health purposes entails the comparison of health outcomes under different treatments or interventions (e.g., comparing the probability of acquiring an infection when vaccinated with the probability of acquiring an infection when not vaccinated). For inferring causality, randomized controlled trials (RCTs), in which individuals are assigned randomly to one of the intervention groups, are recognized as the “gold standard” [1]. When individuals are randomly assigned to an intervention group, the groups are assumed to be exchangeable or “comparable”, meaning that differences in the outcome can be ascribed solely to the exposure of interest [1, 2]. However, it can be of interest to assess the effect of a treatment or intervention in less controlled real-world settings, considering larger populations, obtained by less restrictive criteria for inclusion, to increase the external validity of the study [3]. Further, it is often not ethical or feasible (e.g., because of economic constraints) to perform an RCT. For these purposes, observational studies can be performed, leveraging “real-world” data sources, often obtained through the secondary use of routinely collected health, care and administrative data. When estimating causal effects using observational data, it is essential to consider different potential sources of bias, such as confounding, selection, and information bias, that can appear in natural environments uncontrolled by researchers [1]. The presence of confounders (i.e., variables that influence both the exposure and outcome variable of interest) can result in non-exchangeability of exposure groups, introduce spurious association and, in this way, distort the measured association between exposure and outcome from the causal effect of interest (i.e., differences in the outcome cannot completely be ascribed to the exposure of interest) [1, 2, 4]. Statistical methods, such as confounder adjustment or matching, can be applied to limit confounding bias and to pursue exposure groups that are conditional exchangeable (i.e., comparable) when exchangeability by design (as in an RCT) is not obtained. These methods generally require the availability of detailed patient information. Alternatively, selection bias represents bias introduced by mechanisms for selecting individuals into the analysis. Selection bias can likewise lead to non-exchangeable exposure groups (i.e., compromised internal validity), as well as impaired generalizability of the study results (i.e., external validity) [1, 5]. Lastly, we refer to information bias as a distortion of the measured association resulting from errors in the measurement or classification of variables, such as the exposure, outcome, or covariates in the analysis. Hernán et al. (2022) [6] suggested that specifying a hypothetical RCT that would allow the estimation of the causal effect of interest (a target trial) and emulating this target trial using the available observational data is beneficial for maintaining the elements of an RCT. For example, emulating randomization as specified in the target trial during the analysis may help to reduce the risk of confounding and increase the internal validity of the study [1, 7, 8].

A treatment or intervention can be applied to populations spanning different regions or countries, with the collected real-world observational data often stored decentralized in isolated environments. Integrating and analyzing these data from different locations and institutions, can support public health decision-making by providing more precise and generalizable estimates. A meta-analysis integrating evidence from different independent studies, for example, as maintained by the International Vaccine Access Center (IVAC) on the effectiveness of severe acute respiratory syndrome coronavirus 2 (SARS-CoV-2) vaccine primary series of the online VIEW-hub [9], can be conducted to obtain a pooled effect estimate. However, heterogeneity in the considered confounding factors, criteria for study participant selection, definitions of variables and adopted statistical methods might exist across studies, limiting comparability. When it is of interest to estimate causal effects and compare interventions or public health programs deployed across (particularly, national) borders, using observational sensitive individual-level data, a federated approach (as opposed to an approach using pooled data) can be used. Such a methodology, implying data visiting, allows to approach causal inference in a privacy-preserving and interoperable way, without sharing sensitive data or gathering them in a centralized location. When conducting federated research, interoperability challenges (i.e., obtaining consistent data from distributed data sources, reproducing an analysis, and comparing the results across the data sources), should be addressed. Different layers of interoperability were defined by the European Interoperability Framework (EIF), namely, Legal, Organizational, Semantic and Technical (LOST) interoperability [10, 11]. González-García et al. (2021) [12] presented a methodology and recommendations on how to cope with challenges at the different layers of interoperability when conducting federated research. The current work aims to build upon this pragmatic approach, extending it to a framework amenable to approach causal inference. Previously, technologies or infrastructures for distributed analysis, such as DataSHIELD [13, 14], the Personal Health Train (PHT) [15, 16], and VANTAGE6 [17], have been proposed. However, to the best of our knowledge, guidance on the full methodological process to approach causal inference, including the specification of data requirements and guaranteeing interoperability when being confronted with a causal research question in federated research, is currently lacking.

The BeYond-COVID (BY-COVID) project (2021–2024) is a Horizon Europe funded project aiming to accelerate access to and linkage of SARS-CoV-2, coronavirus disease 2019 (COVID-19) and patient data, and increase preparedness for future pandemics within Europe [18]. The use cases defined within the BY-COVID project are aimed to ensure interoperability across national borders by enabling a federated approach complying with privacy and data protection regulations. This work conceptually describes the proposed methodology and prepares its application to a policy-relevant research question (i.e., investigating the real-world effectiveness of the SARS-CoV-2 primary vaccination program in populations spanning different countries), aiming to facilitate a rapid response in the case of a next pandemic.

## Methods

A methodological framework for federated causal inference research by re-using routinely collected observational data across different regions, was constructed based on the principles of interoperability at Legal (i.e., privacy-by-design), Organizational (i.e., analysis coordination), Semantic (i.e., built upon a common data model) and Technical (i.e., via the distribution of analyses and a reproducible environment) level [11], Open Science (i.e., transparent and accessible processes and knowledge) [19, 20] and international cooperation driven by population-level research questions. The framework expands methodologies to leverage population health data for federated policy-oriented research proposed within the “Information for Action” Joint Action (JA-InfAct) [12, 21] and Population Health Information Research Infrastructure (PHIRI) project [22], allowing it to address causal research questions, through applying existing methodologies (e.g., the use of Directed Acyclic Graphs) and building on literature, experience and expertise.

### The methodological framework

**Fig. 1:**
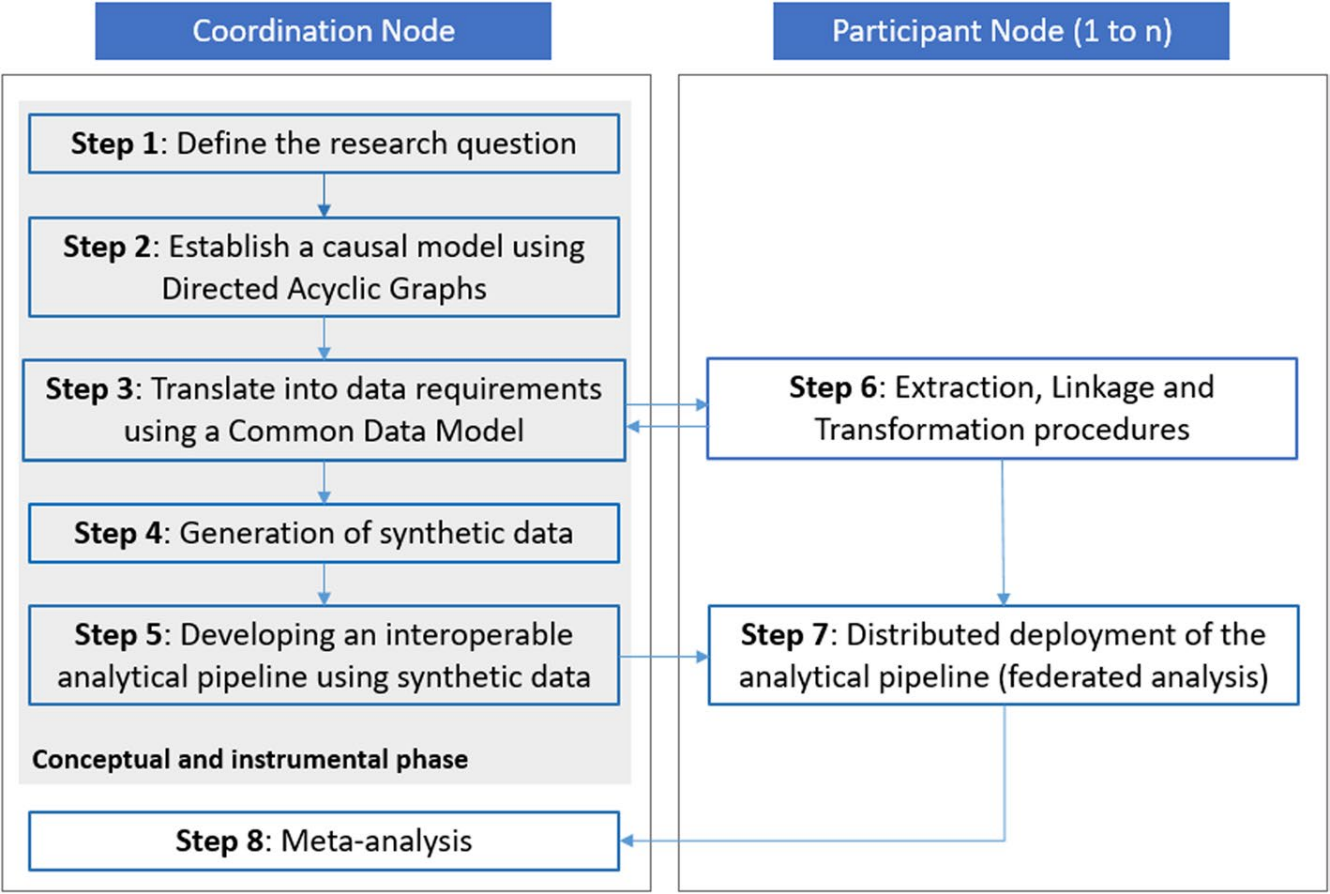
Visual representation of the proposed methodological framework

The developed methodological framework is described in this section. The framework comprises guidelines in the form of the following steps: (1) defining the research question, (2) establishing a causal model using Directed Acyclic Graphs (DAGs), (3) translating the causal model into data requirements using a Common Data Model (CDM), (4) generating synthetic data, supporting script development and testing, (5) developing an interoperable analytical pipeline using synthetic data, (6) extracting, linking, and transforming individual-level data within each node to comply with the CDM specification and information requirements, (7) distributed deployment of the analytical pipeline (i.e., federated analysis), and (8) meta-analysis of the local results (see Fig. 1). Step 1 to 5 are part of a ‘conceptual and instrumental phase’ within the framework and can be conducted without access to real-world data, while steps 6 and 7 involve the extraction, transformation and analysis of real-world data within the jurisdiction of each of the participants to reach step 8 and produce comparable results to inform policy. Going through the steps of the conceptual and instrumental phase of the framework requires profound knowledge about real-world data.

The proposed framework requires close collaboration between a coordinating research team (also referred to as the ‘Coordination Node’) and institutions hosting or being able to acquire access to the required sensitive individual-level data (also referred to as ‘Participant Nodes’), to guarantee organizational interoperability (see Fig. 1). The Coordination Node is responsible for leading the entire process, promoting the collaboration of the participants in the conceptual and instrumental phase of the framework and producing the documentation (i.e., research objects such as the CDM, the synthetic dataset, the analysis scripts of the interoperable analytical pipeline, etc.) supporting the data linkage, preparation and the deployment of the analytical pipeline. In the sections below, a detailed description of each step is provided.

#### Step 1: defining the research question

To start, it is essential to precisely define the research question that the study aims to answer. To guide the formulation of the research question, one can follow the PICO(T) strategy frequently used in clinical research, identifying (1) the patient, problem, population, or cohort of study, (2) the intervention or exposure, (3) the comparison or control, (4) the outcome(s) and, optionally (5) the time frame [23–25]. The research question directly feeds into a causal model, study design and information requirements.

#### Step 2: establishing a causal model using Directed Acyclic Graphs (DAGs)

To estimate the causal effect of interest for the defined exposure-outcome relationship, exchangeable exposure groups should be pursued, thereby emulating randomization of the exposure as in an RCT [26]. Approaching exchangeability requires the adjustment for all known factors confounding the relationship between exposure and outcome. For the identification of these factors, causal models, such as graphical DAGs, can be used as an instrument to collaborate and map conditions to advance towards causal inference. DAGs provide a clear graphical way to identify confounding bias and other potential sources of bias under the described assumptions, and present a way to determine the smallest set of variables to condition on to draw up to a causal association (a ‘minimal sufficient adjustment set’) by using the ‘backdoor criterion’ (i.e., the criterion holds for a set of variables, if all backdoor paths between the exposure and outcome are closed by conditioning on these variables and if none of the variables is a descendent of the exposure) [1]. They map the knowledge and assumptions of researchers about the causal relationship between the exposure and outcome, and give an explicit view on the assumed relationships [27–29]. This way, DAGs increase transparency and facilitate discussion between researchers. The DAGitty web application or corresponding R package ‘dagitty’ can be used to construct and analyze DAGs [30]. Guidance on the construction of DAGs and the identification of a minimal sufficient adjustment set can be found elsewhere [28, 29, 31]. The assumption of ‘no unmeasured confounding’ to identify the causal effect of interest demands appropriately measured confounders and correct statistical inclusion. It is important to consider the possibility of unmeasured or omitted confounding (e.g., due to limitations of surveillance systems or the human understanding of causal relationships respectively) [32].

#### Step 3: translating the causal model into data requirements

Once agreed upon, a causal model should translate the research question into data requirements detailing syntactic and semantic considerations to achieve interoperability and enable sound comparability between the Participant Nodes within the federation. These data requirements are captured in a CDM. A customizable template for building a CDM is available in Additional File 1. All nodes in the DAG (e.g., variables measuring the exposure, outcome and the minimal sufficient adjustment set, as well as variables required to achieve secondary objectives of the study or to perform supplementary or exploratory analyses) should be captured within the model description of the CDM, irrespective of their inclusion in the minimal sufficient adjustment set. Variable labeling must be consistent and follow a pre-specified convention (e.g., Snake Case, Camel Case) [33, 34]. Variable labeling must not hinder the analysis, and therefore the variables should not start with reserved characters or numbers. Furthermore, variable labeling must include information on variable type for easier identification while interactively exploring the data. For example, the following convention can be used: ‘cd’ for categorical variables, ‘nm’ for numerical variables, ‘bl’ for binary/logical variables, ‘dt’ for date variables, and ‘id’ for the primary (and secondary) key of the entity. Each of the variables should be characterized within the model description of the CDM in a detailed manner, including (1) the model entity, (2) the variable label, (2) a description of the variable, (3) the encoding system, (4) the variable format and type, (5) the units of measurement, (6) the requirement level, (7) the variable-level validation rules, (8) the variable properties (observed/calculated), and (9) the possible data sources. The variable format can be expressed differently depending on the data types enabled in each scripting language, however, can commonly be defined as ‘integer’, ‘double’ or ‘float’ for a number, ‘string’ or ‘character’ for an alphanumeric, ‘logical’ or ‘binary’ for TRUE or FALSE and ‘date’ or ‘timestamp’ for a date. The requirement level (i.e., required, recommended or optional) denotes the impact of complete absence of information on that variable on achieving the purposes of the study. In studies aiming to approach causal inference, the required variables in the CDM should correspond to those measuring exposure, outcome and the minimal sufficient adjustment set required to close all backdoor paths identified in the DAG. As such, not having any information (complete missingness) on a variable in the minimal sufficient adjustment set, impedes reaching the study objectives by introducing bias and hindering causal interpretation of obtained estimates. Depending on the context and planned analyses, a variable considered to be required can be allowed to have a certain degree of values missing. Complete missingness of recommended variables could harm the secondary objectives of a study (i.e., planned sensitivity, subgroup analyses, or similar), while complete missingness of optional variables might impede supplementary or explorative analyses. Specifying possible data source(s) and comments are out of the scope of the variable description, but can offer additional information to facilitate the extraction, linkage and transformation procedures, and management of the data at origin during step 6. If different entities (e.g., person, area, test, vaccination dose) are needed to cover the requirements captured in the DAG, a model description per entity should be provided. Further, a variable capturing information on a certain (co)morbidity might demand the specification of crosswalks (i.e., mapping to different classification systems) to ensure the coverage of the definition within different Participant Nodes using different disease classification systems at origin (i.e., semantic interoperability). The data model specification should additionally contain an unambiguous cohort description, including the specification of eligibility criteria of the study population and the start and end date of the study period. Further, in order to make the data model discoverable for other researchers, a structured metadata file should be provided.

#### Step 4: generation of simulated synthetic data

The generation of synthetic data, representing the specifications from the CDM, can be instrumental to develop the interoperable analytical scripts and can serve to exemplify the required data for the federated analysis. Synthetic data can be simulated by simply capturing the technical and syntactic requirements as specified in the CDM and using non-informative mathematical distributions, thereby avoiding exposure of the real sensitive data during the conceptual and instrumental phase of the framework and promoting the development and testing of the analytical scripts while managing the data access application process. Nonetheless, simulated data can be enhanced with expert information on the topic to reflect the expected distributions of the actual data based on published healthcare statistics or prior research. Alternatively, when access to real data is possible and a sufficient degree of anonymization can be assured, synthetic data can be modelled based on the real data (i.e., data driven), preserving its underlying distributions, relationships and statistical properties with the specifications defined in the CDM.

#### Step 5: developing an interoperable analytical pipeline

Once the data requirements are specified and a synthetic dataset is generated, an analytical pipeline for distributed deployment can be developed. The analytics are dependent on the specified research question and can apply different methods to address biases (e.g., adjusting for identified confounders [1, 35], controlling for selection bias [36]) and handle missing data [37–39]. Further, there are various ways to investigate the presence of biases in the results, such as selection and unmeasured or omitted confounder bias, and assess the sensitivity of the results to the applied methods and assumptions in different sensitivity analyses. Nonetheless, irrespective of these specific analytical methods, certain elements common to any federated study should be contained within the pipeline. The first step in the analytical pipeline consists of a comprehensive Data Quality Assessment (DQA), including information on the completeness, uniqueness, and integrity [40]. Next, compliance with the CDM specification should be checked, by testing the input data against a set of data validation rules. Further, descriptive statistics can be produced, providing characteristics of the study population. Population characteristics can be used to improve interpretation of the results and detect potential biases, along with the results from the DQA and validation procedure. Finally, as federated research relies on the distribution of scripts for the analyses and the local deployment and execution of the analyses at each participant’s system, it requires extensive documentation of all functionality and implemented decisions during the development of outputs of the analytical pipeline. All this documentation is required for interpretation of the local outputs, which are later used in the meta-analysis. The analytical pipeline should only produce aggregated results that have lost all sensitive properties, i.e., compliant with disclosure policies.

#### Step 6: extraction, linkage and transformation procedures within the participant nodes

We defined ‘Participant Nodes’ as institutions contributing to the investigation of the research question, hosting or being able to acquire access to individual-level real-world population, health and care data. Each Participant Node is responsible for the data access application process, requesting access to analyze the required data. When access to the data necessary for the research in question is granted, linkage of different data sources needed to comply with the specified data requirements should be performed by the data controllers (i.e., can be the Participant Node or another institution). The Participant Nodes are responsible for processing the data following the guidelines provided by the CDM specification, in this way preparing the data for the analysis. Perfect adherence to the CDM specification cannot always be achieved with the available data, however should be pursued, particularly for the cohort selection criteria, the syntactic model and the required variables.

#### Step 7: distributed deployment of the analytical pipeline

The interoperable analytical pipeline should subsequently be distributed and deployed within a secured processing environment of each Participant Node. It requires as input the linked and transformed data complying with the CDM specification. Adherence of these input data to the CDM should be informed throughout the analytical pipeline through informative errors (i.e., in the event that the input file format is not as expected, or the input file header does not correspond to the expected variables’ names and order), and through the output of the DQA and the validation assessment. The analytical pipeline can be provided as single or multiple scripts implementing the statistical analysis using auditable open-source software or can be containerized (e.g., using a Docker container [41, 42]), providing a fixed environment dealing with system and software dependencies, thus ensuring reproducibility by providing a sandbox that can be deployed and run isolated from the Participant Node’s systems [12, 43]. Containerization also enables easy pipeline distribution as container images can be published in an open repository facilitating versioning and collaborative improvement. Technologies offered by the PHT [15, 16], DataSHIELD [13, 14], and VANTAGE6 [17] can alternatively provide a solution to distribute analysis code to different Participant Nodes.

#### Step 8: Meta-analysis of the local results

To integrate results across different populations, the aggregated non-sensitive statistics produced as local outputs of the analytical pipeline should be pooled by the Coordination Node and a meta-analysis should be performed. By only sharing non-sensitive aggregated results, compliance with General Data Protection Regulation (GDPR) legislation and legal interoperability is ensured. The type of aggregated statistics (e.g., propensity scores, standardized risks, average treatment effects) that are shared and pooled, and the methodology used to integrate these estimates, will depend on the defined research question and should be detailed in the relevant research object (e.g., Statistical Analysis Plan). As indicated previously, some fixed outputs common to any federated study (e.g., documentation of the functionality and implemented decisions during the development of outputs, results from a DQA, validation assessment and descriptive analysis) should also be collected by the Coordination Node, thereby improving the interpretation of the results. In addition to the main results, results from several sensitivity analyses, investigating the presence of biases and sensitivity of the results to certain methods, should be shared with the Coordination Node.

### An illustrative example

The conceptual and instrumental phase (steps 1 to 5) of the proposed methodological framework for federated causal inference (re-)using observational data sources was demonstrated within the BY-COVID project by prototyping a workflow which can be used to assess the real-world effectiveness of SARS-CoV-2 primary vaccination as compared to partial or no vaccination in preventing SARS-CoV-2 infection in populations spanning different countries. In the current manuscript, we showcase the different steps of this initial phase of the methodological framework, preparing for the subsequent implementation of the proposed methodology to respond to a policy-relevant research question. For developing the workflow, only open-source software, such as DAGitty [30], R and DuckDB [44], was used.

## Results

**Fig. 2:**
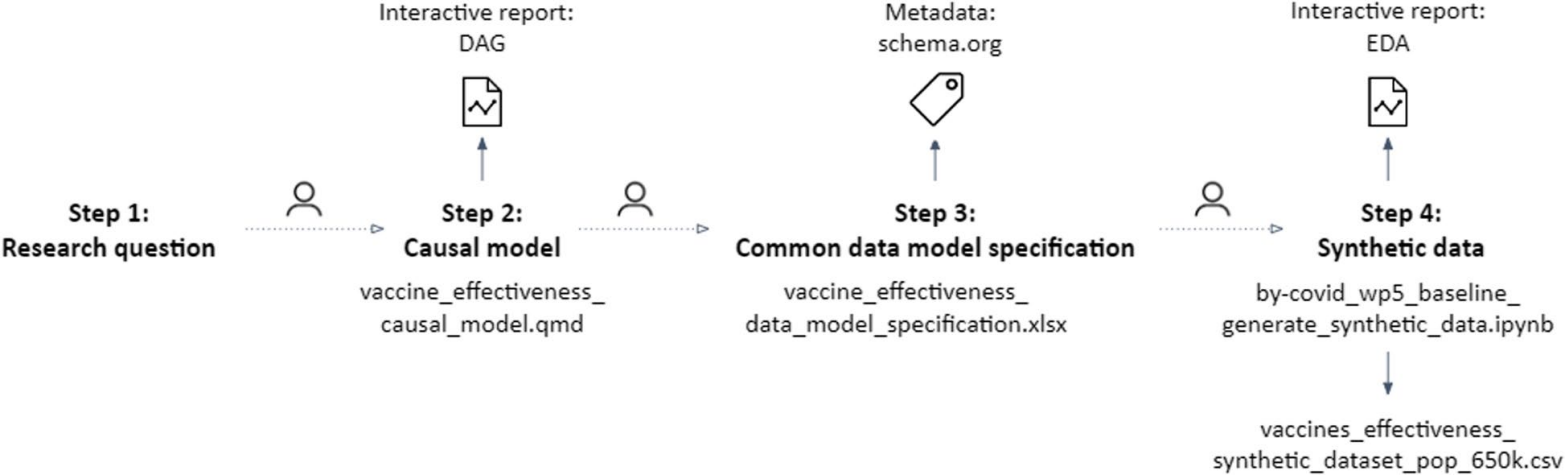
Overview of the executed steps and produced research objects during the implementation of the proposed methodological approach, step 1 to 4, preparing for the assessment of the real-world effectiveness of a primary vaccination schedule as compared to partial or no vaccination in preventing SARS-CoV-2 infection, in populations spanning national borders

Here, we showcase the conceptual and instrumental phase of the proposed methodological approach as established within the BY-COVID project’s use case. Steps 1 to 4 and the related research objects are presented in Fig. 2.

**Fig. 3:**
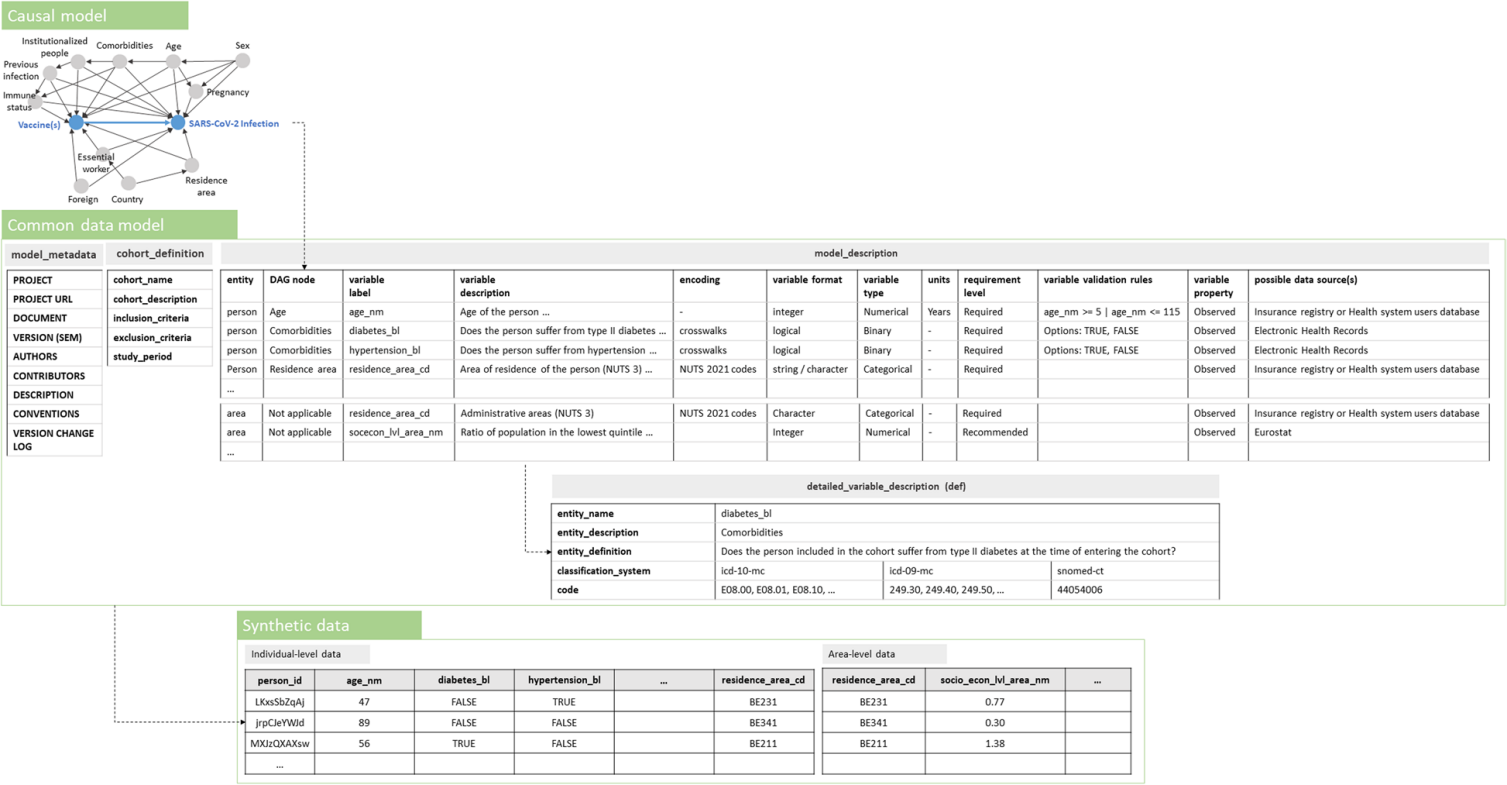
The causal model (using a DAG), Common Data Model (CDM) and synthetic data, and how they relate to each other. The DAG, capturing assumptions on factors and relationships when assessing the real-world effectiveness of a primary vaccination schedule as compared to partial or no vaccination in preventing SARS-CoV-2 infection in populations spanning national borders, is visualized. The structure of the CDM and synthetic data, as constructed based on the drafted causal model, is presented

To start, a research question was defined following the PICOT strategy (*step 1*), namely, we aim to assess the real-world effectiveness of a primary vaccination schedule as compared to partial or no vaccination in preventing SARS-CoV-2 infection, in populations spanning national borders [45], with the purpose of evaluating the effectiveness of the basic vaccination campaign. Individuals (age 5 to 115, resident of the participating country) vaccinated with at least one dose of the SARS-CoV-2 vaccine (any of the available brands), or eligible to be vaccinated and with a documented positive diagnosis (irrespective of the type of test) for a SARS-CoV-2 infection during the data extraction period, are eligible for inclusion. Individuals with a documented confirmed infection before completing the primary vaccination schedule (i.e., enrolment), or before January 1, 2021 (SARS-CoV-2 vaccine roll-out) for those not having completed a primary vaccination schedule (controls), will be excluded from the study population. A DAG corresponding to the research question was produced and a ‘minimal sufficient adjustment’ set was identified using the DAGitty web application [30] (*step 2*). Nodes and edges within the DAG were defined as assumptions based on relationships described in the literature. Once an initial DAG was drafted, field experts participating in the BY-COVID project were invited to discuss and adapt the captured assumptions where needed. The following DAG nodes present a minimal sufficient adjustment set, conditional on the assumptions that were made: Age, Comorbidities, Country, Essential worker, Foreign, Immune status, Institutionalized people, Pregnancy, Previous infection, Residence area and Sex. A Quarto notebook was developed (see file vaccine_effectiveness_causal_model_v.1.1.0.qmd or a later version as available in the Zenodo publication [46]), generating an interactive report that visualizes the DAG together with information on the research project and the identified minimal sufficient adjustment set. Figure 3, displaying the constructed DAG, illustrates how the digital objects that are consecutively produced during the conceptual and instrumental phase of the proposed methodological approach relate to each other. The DAG was translated into data requirements using a CDM (*step 3*), operationalizing all the nodes in the DAG. Additional individual- and area-level variables were specified to achieve secondary objectives of the study and to perform supplementary or exploratory analysis (e.g., variables variant_cd or socecon_lvl_area_nm). Variables were labeled following the Snake Case naming convention. The CDM consists of an Excel file (see file vaccine_effectiveness_data_model_specification_v.1.1.0.xlsx or a later version as available in the Zenodo publication [46]) including a tab with a cohort description, a tab with the model description (characterization of variables), and tabs with a detailed description of certain variables (e.g., comorbidities requiring crosswalks). International classification systems were used when specifying the required encoding of variables and when specifying crosswalks (e.g., IDC-10, IDC-9, and SNOMED-CT for classifying comorbidities). The structure of the CDM is presented within Fig. 3. Compliance to the requirements captured in this CDM can be achieved by performing a full join of the registered individuals in the COVID-19 cases and vaccination datasets and the individual-level linkage to additional data sources, such as patient administrative information (e.g., from insurance registries, health system users-databases, and mortality registration data) and information on patient comorbidities (e.g., from Electronic Health Records) within the Participant Nodes (which is foreseen in *step 6*). A metadata description of the CDM was provided using the Schema.org vocabulary.

A synthetic dataset (see file vaccines_effectiveness_synthetic_dataset_pop_650k_v.1.1.0.csv or a later version as available in the Zenodo publication [46]) was subsequently generated (*step 4*), translating the CDM specification into a Python script parameterized to simulate data, considering several known population-level parameters for the COVID epidemic waves (see file by-covid_wp5_baseline_ generate_synthetic_data_v.1.1.0.ipynb or a later version as available in the Zenodo publication [46]). Within this Python script we made use of the Python package ‘Faker’ [47]. An exploratory data analysis (EDA) was performed on the synthetic data, exploring different features of the data (i.e., type inference, alerts, uniqueness, outlier values, missing data, univariate analysis) to assess its compliance with the CDM (see file vaccine_effectiveness_synthetic_dataset_eda_v.1.1.0.html or a later version as available in the Zenodo publication [46]). Based on the EDA, we observe that the generated synthetic data correctly capture the syntactic and technical specifications provided by the CDM. Particularly, the variables in the synthetic data, their labels, encoding, format and type match those specified in the CDM. Variables corresponding to nodes in the minimal sufficient adjustment set have no (e.g., age_nm and sex_cd) or a limited proportion of (e.g., residence_area_cd with 2% missing) missing values.

**Fig. 4:**
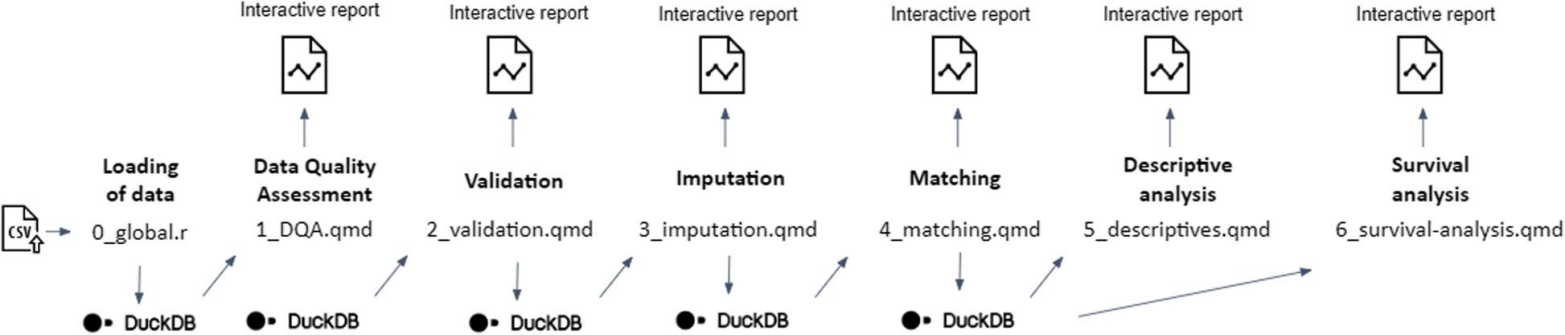
Graphical overview of the developed analytical pipeline, consisting of different subsequent modules, each producing an interactive report. Implementation of step 5 of the proposed methodological approach to assess the real-world effectiveness of a primary vaccination schedule as compared to partial or no vaccination in preventing SARS-CoV-2 infection, in populations spanning national borders

Subsequently, an analytical pipeline was developed and tested with the support of the synthetic data (*step 5*) using the R statistical programming language as sequential Quarto documents (.qmd files) reflecting and reporting the outputs of different modules: thus, (1) DQA of the original input data, (2) validation (i.e., applying logic validation rules) of the original input data to check compliance with the CDM, (3) imputation of missing data where required, (4) iterative matching of the exposed to unexposed individuals and a balance assessment of the matched population, (5) a descriptive analysis of the matched and unmatched study population, and (6) a survival analysis in the matched study population (see the GitHub repository for methodological details [48]). A graphical overview of the analytical pipeline is presented in Fig. 4. Each module of the analysis produces an interactive report, including documentation allowing to trace back to decisions made along the way and interpreting the results. The DQA script (1_DQA.qmd) embedded in the analytical pipeline was roughly inspired by the data profiling produced by the ydata-profiling Python library [49, 50], usually considered an industry standard for Exploratory Data Analysis in Python, and output contains descriptive dataset statistics and a basic profile of the dataset as a whole (among others containing a variable count, a row count, a basic missing data profile, and some alerts regarding the cardinality, missingness or anomaly of certain variables). The DQA also contains a univariate descriptive analysis of each variable in the dataset, providing summary statistics, information on the categories of the categorical variables, and basic information on the distribution of the continuous variables. Validation of the data, i.e., checking compliance of the data to validation rules captured in the CDM and exclusion of non-compliant data for further analysis, is captured within the script 2_validation.qmd. An algorithm capturing decisions on how to deal with missing values in the imported dataset was developed in script 3_imputation.qmd, implementing the imputation of values, listwise deletion or exclusion of matching variables depending on the characteristics of the data. The script 4_matching.qmd implements the daily matching of exposed to unexposed individuals on variables corresponding to nodes in the minimal sufficient adjustment set following the causal model, thereby attempting to close non-causal backdoor paths and limit bias. After describing the study population and providing crude estimates in script 5_descriptives.qmd, a survival analysis is captured within script 6_survival-analysis.qmd, visualizing survival over time by producing Kaplan-Meier curves and estimating the average treatment effect (ATE). A detailed documentation of the statistical methods, as well as a README file guiding users on the script deployment, accompanies the statistical scripts in the GitHub repository [48]. DuckDB, a lightweight database system, is used to increase the speed of running the workflow by enhancing performance when dealing with large amounts of data and complex analytical queries.

The DAG, CDM and synthetic dataset, together with all supporting research objects (see Fig. 2), were published on Zenodo [46]. Further, the latter plus additional digital research objects produced along the way (i.e., a study protocol [45], a data management plan (DMP) [51], and an interoperable analytical pipeline) were collected together in a GitHub Repository [48]. A Research Object Crate (RO-Crate) [52] was generated to package these objects together with their metadata and specified relationships, acting in accordance with the FAIR (Findable, Accessible, Interoperable, and Reusable) data principles [53]. This way, the digital research objects are persisting and shared with the wider community, and as such remain available for feedback from field experts.

## Discussion

We present a methodological framework, providing a systematic approach to address policy-relevant causal research questions based on federated cross-national sensitive observational data in a privacy-preserving way and addressing challenges at different layers of interoperability. In this way, the current manuscript aims to provide guidance on the full methodological process to approach causal inference in federated research, which is currently lacking. This approach facilitates the comparison and integration of causal estimates obtained from distributed analyses through approaching homogeneity in the considered confounders, criteria for study participant selection, definitions of variables and adopted statistical methods. The conceptual and instrumental phase of the proposed methodological framework, consisting of different consecutive steps, was successfully demonstrated within a Use Case of the European BY-COVID project, thereby preparing the subsequent assessment of SARS-CoV-2 vaccine effectiveness in preventing infection in a population crossing national borders and prototyping a workflow that is standard for causal population health research. By going through these steps, different challenges and limitations of the proposed methodology were identified. These challenges, together with recommendations on how to address them, are described in the following sections.

### Challenges and recommendations in addressing the emulation of an RCT using federated sensitive cross-border observational data

Estimation of the causal effect of interest can be approached by emulating an RCT, as suggested by Hernán et al. (2022) [6], thereby mimicking randomization by conditioning on confounders and controlling for other potential biases. Crucial in this process is the identification of the set of confounders for which adjustment during the analysis is required to approach causal association under the assumptions captured in the causal model. However, this is a non-trivial task given that not all underlying mechanisms may be known [54]. Theoretical identification of confounding paths and other biases was based on the assumptions of the researchers on the data-generating processes (e.g., method of participant selection and variable measurement, or relationships between variables), which were in turn built upon available theory and expert knowledge at the time of analysis. Further, detailed patient information is required to adjust for the identified confounders and the necessary data might not be available in all Participant Nodes. When this is the case, it may result in the presence of residual confounding bias in the obtained causal effect estimates, hampering both the internal and external validity of the study [55–57]. Moreover, taking into account potential biases when conducting causal inference using observational data results in complex analytical procedures which generally demand several human decision points. This raises the question whether analytical procedures to approach a complex causal research question can be fully automated. Sensitivity analyses can be implemented to assess the presence of biases, and sensitivity of the results to the methods implemented for confounder adjustment.

**Recommendation 1** The use of DAGs is recommended to build causal models, transparently displaying assumptions, aiding the identification of a minimal sufficient adjustment set (i.e., set variables for which you are required to adjust to estimate the causal effect of interest under the described assumptions), and in this way feeding into the specification of data requirements. DAGs are easy-to-use graphical instruments, facilitating explicit specification of assumptions. The DAGitty web application provides a practical tool to create and edit DAGs, and to identify the minimal sufficient adjustment set.

**Recommendation 2** By enabling field experts to provide feedback on the assumptions and iteratively updating the DAG when new information becomes available (i.e., building the causal model as a collaborative effort), theoretical identification of confounders and other sources of bias can be optimized. As such, we encourage making the constructed causal model publicly available, working on collaborative platforms (e.g., GitHub), and providing occasions (e.g., workshops) for field experts to evaluate it.

**Recommendation 3** Acknowledging the limitations to a causal interpretation of the results can be addressed by including an assessment of the data quality at each step of the analytical process. In addition to a general DQA and validation assessment, quality evaluations specific to the performed analytics can be performed. For example, when matching based on identified confounders is performed to obtain comparable intervention groups, an assessment of covariate balance is conducted and reported thereafter. In addition, every point of automated decision making should be documented in the output of the analytical pipeline to allow for a meaningful interpretation of the obtained results. For instance, automated decisions on whether or not to impute missing values or perform listwise deletion, are reported in an interactive report. Quarto [58], an open-source tool, provides an efficient way to produce rich interactive outputs, registering the latter information.

### Challenges and recommendations on the different layers of interoperability (LOST) when conducting federated causal research

#### Legal interoperability

Legal constraints based on privacy and data protection regulations (GDPR) can block the re-use of sensitive personal data for population health research across (national) borders. Implementing a federated analysis approach where sensitive data stays under the jurisdiction and governance of data holders (i.e., data visiting principle) offers a solution. However, some steps in the proposed methodology may still pose legal challenges. Data-driven methods for developing synthetic data [59] might give rise to concerns of re-identification and require access of the Coordination Node to real data. Further, for individual Participant Nodes to comply with the CDM specification, linkage of and access to the required data sources for the research in question should be authorized. Access to sensitive real-world health data is in many European countries granted by an authorizing body, such as the national Data Protection Authority (DPA) or Research Ethics Committee (REC), based on the evaluation of a study protocol and data management plan. However, no standard process for applying for data access is available at this time in Europe, preventing the use of a uniform approach.

**Recommendation 4** Building a CDM, specifying data requirements, is recommended to comply with the principles of data minimization. In this way, we can limit the collection of sensitive information to what is strictly relevant and necessary for the purpose. By including a requirement level in the description of variables specified in the CDM, it can be indicated which variables are essential to close backdoor paths identified in the DAG.

**Recommendation 5** To facilitate a rapid data access application process, it is recommended for the Coordination Node to provide the Participant Nodes with the necessary tools, i.e., produce and share a comprehensive study protocol and give guidance for the development of a research DMP. A study protocol provides a plan of action and contains among others the study objectives and planned methodology for conducting the study. The study protocol additionally facilitates compliance with purpose minimization principles. Guidelines for writing a scientific study protocol can be consulted elsewhere [60]. For the development and publishing of a DMP, use of Argos’ services [61, 62] can be recommended.

**Recommendation 6** As data-driven methods (i.e., based on real-world data) for developing synthetic data might have some weaknesses in terms of legal interoperability, we advise the manual development of synthetic data, without requiring access to real data [59], capturing the structure, syntactic and semantic requirements as specified in the CDM and reflecting true distributions by using known population-level parameters.

#### Organizational interoperability

To achieve organizational interoperability and reach common goals, organizations should define and align responsibilities, processes, and expectations [63, 64]. A diversity of theoretical backgrounds of researchers in the federated network was observed, resulting in the need for building a common ground. Further, unambiguously defining data requirements, which is essential to obtain uniform data across different federations, was additionally found to be a difficult task. An approach to collaboratively address the causal research question was required.

**Recommendation 7** Clearly assigning responsibilities (i.e., allocating the role of Coordination versus Participant Node), documenting processes and exchanging relevant information (i.e., publishing and sharing a research protocol, DMP, and digital research objects), and facilitating interactions within the federated network (e.g., using a collaboration platform) is recommended to achieve organizational interoperability. It is the role of the Coordination Node to supervise and synchronize the activities executed by the Participant Nodes, and to provide information and support to establish a common knowledge on the process and required involvement. For example, in the demonstrated phase of the framework a theoretical overview on DAGs was given by the Coordination Node to participating partners in the form of a workshop. Regular contact with the Participant Nodes is required throughout the process, but more intensely when agreeing on the research question and defining the CDM specification. This allows the Participant Nodes to put forward ambiguities and requests for clarification. Transparency of the process, making research objects produced in every step openly available, is recommended to enhance trust and allow for providing expert-knowledge and user-based feedback. Use of a collaborative and version control platform, such as GitHub, enables collaboration with several partners.

#### Semantic interoperability

Semantic interoperability indicates the consistency in meaning of exchanged data among organizations [65], enabling the interpretation of data independently of the partner involved. When working across borders, different Participant Nodes can have distinct codebooks and use different classification systems. Mapping a definition to different classification systems and identifying intersections between these classification systems (i.e., specifying crosswalks), is not always straightforward. Further, definitions of variables and cohorts can be ambiguous and open to interpretation.

**Recommendation 8** The construction of a CDM is recommended to ensure a uniform syntactic structure (i.e., format and grammar) and meaning (i.e., semantics) of elements of the distributed data used to address the specified cross-border research question. To improve compliance with data requirements and consistency between distributed datasets, involving collaborators within the nodes in reviewing the specifications captured in the CDM is recommended. Based on this evaluation, ambiguities can be eliminated and the specification of crosswalks, mapping definitions to different classification systems, can be optimized.

#### Technical interoperability

A critical part of deploying a reproducible analytical pipeline, is dealing with dependencies of the pipeline, ensuring consistent deployment independent from the system in which it is executed, and in this way ensuring technical interoperability. Packaging the analytical pipeline created in the prototyped workflow within a container (i.e., an isolated portable execution environment including analytical code), such as a Docker container, presents a way for easy transmission of scripts, easy management of dependencies and allows for consistent execution of the analyses in different premises, decoupled from the local system. However, use of container technology might not be feasible due to a lack of support for Docker within certain operating systems or other organizational barriers to deployment. Further, the functioning of the analytical pipeline code relies heavily on the ability of the Participant Nodes to comply with the CDM specification. When the input data of the analytical pipeline does not conform with the specified semantic and syntactic requirements, the process will fail before generating the required output statistics. Efficiency challenges were also encountered when inferring causality for the entire population of a country or region, requiring the handling of large volumes of data.

**Recommendation 9** When transferring and deploying an interoperable analytical pipeline, the use of existing technological solutions to deal with the required dependencies and allowing deployment of the analytical process consistently and independently from the local execution system (e.g., Docker containers), is recommended. When deploying a container is not feasible within a Participant Node’s system, several alternative strategies can be adopted, such as deploying the Docker within a virtual machine, deploying the Docker container within a research environment provided by a trusted third party, or manual installation of the required dependencies and manual execution of the analysis scripts.

**Recommendation 10** To facilitate rapid deployment in the Participant Nodes, it is recommended to provide users deploying the analytical pipeline with feedback based on error logs when compliance with the structure and syntactic requirements captured in the CDM is lacking for the input data and the process is failing. Further, it is recommended to check the distributed input data against a set of logical validation rules, examining compliance to the specifications captured in the CDM. This can be implemented as one of the sequential steps in the analytical pipeline.

**Recommendation 11** To deal with efficiency challenges when handling large volumes of data, it can be recommended to implement efficient programming strategies [66, 67], to parallelize heavy and repetitive computations where this increases throughput, and work with data management solutions, such as DuckDB [44].

### Challenges and recommendations related to the reuse of digital objects

There is an increasing demand for researchers to document and share the data and research objects supporting their scientific conclusions, to increase transparency, facilitate collaboration, and allow subsequent replication, integration and reuse by the community [53]. In public health, this can be essential in the response to emerging public health threats (e.g., a pandemic). However, processes might not always be well documented, researchers might not be aware of the benefits of sharing their data or research objects (e.g., avoiding duplication, greater visibility), or potentially can’t locate appropriate repositories.

**Recommendation 12** To facilitate the exchange and reuse of the digital research objects of the workflow and in this way enabling an accelerated response in the case of a new pandemic, it is recommended to publish these objects following the FAIR principles. To make your object Findable, rich metadata should be provided. Making the digital objects Accessible, means that they have to be retrievable when access is allowed. For this purpose, the objects can be shared with rich metadata in open repositories like Zenodo. In the context of an interconnected workflow, RO-Crate [52] provides an alternative approach to package research objects together with their metadata, allowing the indication of relationships between entities. Further, the objects should be made Interoperable, by using standards and controlled vocabularies, and Reusable, by providing clear documentation (e.g., a README) [53].

## Conclusion and future perspectives

The proposed methodological framework provides guidance in the form of a systematic approach to address federated cross-national causal research questions in a privacy-preserving way, while tackling challenges at different layers of interoperability. Additionally, the conceptual and instrumental phase of the methodological framework was demonstrated in the current work, thereby prototyping a standard workflow for causal population health research. Describing the methodological framework, publishing the produced research objects (e.g., causal model, CDM and synthetic data) and prototyping a workflow using open-source tools available for reuse, allows researchers to respond more rapidly to newly emerging public health research questions and in this way contributes to pandemic preparedness. Future planned work in the context of the BY-COVID project entails the implementation of the proposed methodology and the actual assessment of SARS-CoV-2 vaccine effectiveness in preventing infection in a population crossing national borders. This proof-of-concept will evaluate the value of the proposed framework in terms of drawing conclusions on causality for the specified research question, the linkage of heterogeneous data sources and data transformation by the Participant Nodes to comply with the specified data requirements, the deployment of the developed analytical pipeline in a distributed manner across different Participant Nodes, and the pooling of these results for a meta-analysis. Further research is needed to test the implications of the implementation of alternative statistical methods for causal inference using a federated research approach, although the framework enables the use of any method currently available. Upgrading the proposed methodological framework and applying it to new policy-relevant questions in emerging public and population health issues can be considered an important research priority in the field of federated causal inference.

### Supplementary Information

**Additional file 1. **

**Additional file 2.**

## Data Availability

The research objects generated during the current study (i.e., a study protocol, a data management plan, a causal model, a common data model, a synthetic dataset, an interoperable analytical pipeline) are available from Zenodo [39] and GitHub, https://w3id.org/ro/doi/10.5281/zenodo.6913045.

## Abbreviations

RCT: Randomized Controlled Trial
IVAC: International Vaccine Access Center
SARS-CoV-2: severe acute respiratory syndrome coronavirus 2
EIF: European Interoperability Framework
LOST: Legal, Organizational, Semantic and Technical
ATE: Average Treatment Effect
BY-COVID: BeYond-COVID
COVID-19: coronavirus disease 2019
DAG: Directed Acyclic Graphs
CDM: Common Data Model
PICO(T): Patient/Problem/Population, Intervention, Comparison/Control, Outcome(s) (and Time frame)
GDPR: General Data Protection Regulation
EDA: Exploratory Data Analysis
DQA: Data Quality Assessment
IACS: Instituto Aragonés de Ciencias de la Salud
DMP: Data Management Plan
RO-Crate: Research Object Crate
FAIR: Findable, Accessible, Interoperable, and Reusable
DPA: Data Protection Authority
REC: Research Ethics Committee
PHT: Personal Health Train
JA-InfAct: "Information for Action" Joint Action
PHIRI: Population Health Information Research Infrastructure

## References

[1] Hernán MA, Robins JM (2020). Causal inference: what if.

[2] Greenland S, Robins JM (2009). Identifiability, exchangeability and confounding revisited. Epidemiol Perspect Innov.

[3] Listl S, Jürges H, Watt RG (2016). Causal inference from observational data. Commun Dent Oral Epidemiol.

[4] Pearce N, Lawlor DA (2016). Causal inference—so much more than statistics. Int J Epidemiol.

[5] Hernán MA, Hernández-Díaz S, Robins JM (2004). A structural approach to selection bias. Epidemiology.

[6] Hernán MA, Wang W, Leaf DE (2022). Target trial emulation: a framework for causal inference from observational data. JAMA.

[7] Hernán MA, Robins JM (2006). Estimating causal effects from epidemiological data. J Epidemiol Community Health.

[8] Glass TA, Goodman SN, Hernán MA, Samet JM (2013). Causal inference in public health. Annu Rev Public Health.

[9] International Vaccine Access Center (IVAC). VIEW-hub. 2023. Available from: https://view-hub.org/covid-19/effectiveness-studies. Cited 2023 Feb 13.

[10] Directorate-General for Informatics (European Commission). New European interoperability framework: promoting seamless services and data flows for European public administrations. Publications Office of the European Union; 2017. Available from: https://data.europa.eu/doi/10.2799/78681. Cited 2023 Mar 1.

[11] Croatian Institute of Public Health (CIPH). Instituto Aragonés de Ciencias de la Salud (IACS). LOST* and found: Report on interoperability landscape in Europe. p. 1–55. Available from: https://www.inf-act.eu/sites/inf-act.eu/files/2020-11/D10.1.pdf.

[12] González-García J, Estupiñán-Romero F, Tellería-Orriols C, González-Galindo J, Palmieri L, Faragalli A, González-García J, Estupiñán-Romero F, Tellería-Orriols C, González-Galindo J, Palmieri L, Faragalli A, Pristās I, Vuković J, Misinš J, Zile I, Bernal-Delgado E, Unim B, Carle F, Gesuita R, Ivanković D, Brkić M, Dimnjaković J, Lyons J, Lyons R, Ors Z, Zaletel M, Nogueira P, Lapão LV, Haaheim H, Bogaert P, Abboud L, van Oyen H (2021). Coping with interoperability in the development of a federated research infrastructure: achievements, challenges and recommendations from the JA-InfAct. Arch Public Health.

[13] Wolfson M, Wallace SE, Masca N, Rowe G, Sheehan NA, Ferretti V (2010). DataSHIELD: resolving a conflict in contemporary bioscience—performing a pooled analysis of individual-level data without sharing the data. Int J Epidemiol.

[14] Gaye A, Marcon Y, Isaeva J, LaFlamme P, Turner A, Jones EM, Gaye A, Marcon Y, Isaeva J, LaFlamme P, Turner A, Jones EM, Minion J, Boyd AW, Newby CJ, Nuotio M-L, Wilson R, Butters O, Murtagh B, Demir I, Doiron D, Giepmans L, Wallace SE, Budin-Ljøsne I, Oliver Schmidt C, Boffetta P, Boniol M, Bota M, Carter KW, deKlerk N, Dibben C, Francis RW, Hiekkalinna T, Hveem K, Kvaløy K, Millar S, Perry IJ, Peters A, Phillips CM, Popham F, Raab G, Reischl E, Sheehan N, Waldenberger M, Perola M, van den Heuvel E, Macleod J, Knoppers BM, Stolk RP, Fortier I, Harris JR, Woffenbuttel BHR, Murtagh MJ, Ferretti V, Burton PR (2014). DataSHIELD: taking the analysis to the data, not the data to the analysis. Int J Epidemiol.

[15] Attema T, Worm D. Technological breakthrough finally, a privacy-friendly way to harness data. 2021. Available from: http://resolver.tudelft.nl/uuid:8002b966-7bba-427c-b343-56326c1a587b. Cited 2023 Sep 5.

[16] Beyan O, Choudhury A, van Soest J, Kohlbacher O, Zimmermann L, Stenzhorn H (2020). Distributed analytics on sensitive medical data: the personal health train. Data Intell.

[17] Moncada-Torres A, Martin F, Sieswerda M, Van Soest J, Geleijnse G (2021). VANTAGE6: an open source priVAcy preserviNg federaTed leArninG infrastructurE for Secure Insight eXchange. AMIA Annu Symp Proc.

[18] BY-COVID. Available from: https://by-covid.org/. Cited 2023 Mar 30.

[19] Spellman BA, Gilbert EA, Corker KS. Open Science. Stevens’ Handbook of Experimental Psychology and Cognitive Neuroscience. John Wiley & Sons, Ltd; 2018. p. 1–47. Available from: https://onlinelibrary.wiley.com/doi/abs/10.1002/9781119170174.epcn519. Cited 2023 May 16.

[20] Foster ED, Deardorff A (2017). Open Science Framework (OSF). J Med Libr Assoc.

[21] Abboud LA, Bogaert P, Fehr A, Urbanski D, Tolonen H, Noguer-Zambran I (2018). The new joint action on health information: information for action (InfAct)!. Eur J Pub Health.

[22] Bogaert P, Schutte N (2021). Towards a population health information research infrastructure. Eur J Pub Health.

[23] Nishikawa-Pacher A (2022). Research questions with PICO: a universal mnemonic. Publications.

[24] Riva JJ, Malik KMP, Burnie SJ, Endicott AR, Busse JW (2012). What is your research question? An introduction to the PICOT format for clinicians. J Can Chiropr Assoc.

[25] Lira RPC, Rocha EM (2019). PICOT: imprescriptible items in a clinical research question. Arq Bras Oftalmol.

[26] Hernán MA, Robins JM (2016). Using big data to emulate a target trial when a randomized trial is not available. Am J Epidemiol.

[27] Staplin N, Herrington WG, Judge PK, Reith CA, Haynes R, Landray MJ, Staplin N, Herrington WG, Judge PK, Reith CA, Haynes R, Landray MJ, Baigent C, Emberson J (2017). Use of causal diagrams to inform the design and interpretation of observational studies: an example from the Study of Heart and Renal Protection (SHARP). CJASN.

[28] Suzuki E, Shinozaki T, Yamamoto E (2020). Causal diagrams: pitfalls and tips. J Epidemiol.

[29] Tennant PWG, Murray EJ, Arnold KF, Berrie L, Fox MP, Gadd SC, Tennant PWG, Murray EJ, Arnold KF, Berrie L, Fox MP, Gadd SC, Harrison WJ, Keeble C, Ranker LR, Textor J, Tomova GD, Gilthorpe MS, Ellison GTH (2021). Use of directed acyclic graphs (DAGs) to identify confounders in applied health research: review and recommendations. Int J Epidemiol.

[30] Textor J, van der Zander B, Gilthorpe MS, Liskiewicz M, Ellison GT (2016). Robust causal inference using directed acyclic graphs: the R package “dagitty”. Int J Epidemiol.

[31] Digitale JC, Martin JN, Glymour MM. Tutorial on directed acyclic graphs. J Clin Epidemiol. 2021. Available from: https://www.sciencedirect.com/science/article/pii/S0895435621002407. Cited 2021 Nov 16. 10.1016/j.jclinepi.2021.08.001 PMC8821727 34371103

[32] Kasza J, Wolfe R, Schuster T (2017). Assessing the impact of unmeasured confounding for binary outcomes using confounding functions. Int J Epidemiol.

[33] Using eye tracking to study variable naming conventions and their effect on code readability. Available from: https://www.diva-portal.org/smash/record.jsf?pid=diva2%3A1337810&dswid=1132. Cited 2023 Sep 8.

[34] DiLeo C, DiLeo C (2019). Naming things. Clean ruby: a guide to crafting better code for rubyists.

[35] Li L, Kleinman K, Gillman MW (2014). A comparison of confounding adjustment methods with an application to early life determinants of childhood obesity. J Dev Orig Health Dis.

[36] Bareinboim E, Pearl J. Controlling Selection Bias in Causal Inference. Proceedings of the Fifteenth International Conference on Artificial Intelligence and Statistics. PMLR; 2012. p. 100–8. Available from: https://proceedings.mlr.press/v22/bareinboim12.html. Cited 2023 Jul 24.

[37] Kang H (2013). The prevention and handling of the missing data. Korean J Anesthesiol.

[38] Haukoos JS, Newgard CD (2007). Advanced statistics: missing data in clinical research—Part 1: an introduction and conceptual framework. Acad Emerg Med.

[39] Rubin DB, Little RJA. Statistical analysis with missing data, third edition. Hoboken: Wiley; 2019.

[40] Cai L, Zhu Y (2015). The challenges of data quality and data quality assessment in the big data era. Data Sci Jour.

[41] Bashari Rad B, Bhatti H, Ahmadi M (2017). An introduction to docker and analysis of its performance. IJCSNS Int J Comput Sci Netw Secur.

[42] Boettiger C (2015). An introduction to docker for reproducible research. SIGOPS Oper Syst Rev.

[43] Piccolo SR, Frampton MB (2016). Tools and techniques for computational reproducibility. GigaScience.

[44] Raasveldt M, Mühleisen H. DuckDB: an embeddable analytical database. Proceedings of the 2019 international conference on management of data. New York: association for computing machinery; 2019;1981–4. 10.1145/3299869.3320212. Cited 2023 May 9.

[45] Meurisse M, Van Goethem N, Estupiñán-Romero F, González-Galindo J, Royo-Sierra S, Martínez-Lizaga N et al. BY-COVID - WP5 - baseline use case: COVID-19 vaccine effectiveness assessment - study protocol. 2023. Available from: https://zenodo.org/record/7560731. Cited 2023 Jan 31.

[46] Estupiñán-Romero F, Van Goethem N, Meurisse M, González-Galindo J, Bernal-Delgado E. BY-COVID - WP5 - Baseline Use Case: SARS-CoV-2 vaccine effectiveness assessment - Common Data Model Specification. 2023. Available from: https://zenodo.org/record/7572373. Cited 2023 Feb 22.

[47] Faraglia D. Welcome to Faker’s documentation! — Faker 18.13.0 documentation. Available from: https://faker.readthedocs.io/en/master/. Cited 2023 Sep 12.

[48] Meurisse M, Estupiñán-Romero F, Van Goethem N, González-Galindo J, Royo-Sierra S, Bernal-Delgado E. BY-COVID - WP5 - Baseline Use Case: SARS-CoV-2 vaccine effectiveness assessment. BY-COVID Project; 2023. 10.5281/zenodo.6913045. Cited 2023 Apr 26.

[49] ydata-profiling. YData. 2023. Available from: https://github.com/ydataai/ydata-profiling. Cited 2023 Sep 13.

[50] Welcome - YData profiling. Available from: https://docs.profiling.ydata.ai/4.5/. Cited 2023 Sep 13.

[51] Martínez-Lizaga N, Meurisse M, Estupiñan-Romero F, Goethem NV, Bernal-Delgado E. BY-COVID - WP5 - baseline use case: COVID-19 vaccine effectiveness assessment - data management plan. 2023. Available from: https://zenodo.org/record/7625784. Cited 2023 May 2.

[52] Sefton P, Ó Carragáin E, Soiland-Reyes S, Corcho O, Garijo D, Palma R et al. RO-crate metadata specification 1.1.3. 2023. Available from: https://zenodo.org/record/7867028. Cited 2023 May 3.

[53] Wilkinson MD, Dumontier M, Aalbersberg IJ, Appleton G, Axton M, Baak A, Wilkinson MD, Dumontier M, Aalbersberg IJ, Appleton G, Axton M, Baak A, Blomberg N, Boiten J-W, da Silva Santos LB, Bourne PE, Bouwman J, Brookes AJ, Clark T, Crosas M, Dillo I, Dumon O, Edmunds S, Evelo CT, Finkers R, Gonzalez-Beltran A, Gray AJG, Groth P, Goble C, Grethe JS, Heringa J, ’t Hoen PAC, Hooft R, Kuhn T, Kok R, Kok J, Lusher SJ, Martone ME, Mons A, Packer AL, Persson B, Rocca-Serra P, Roos M, van Schaik R, Sansone S-A, Schultes E, Sengstag T, Slater T, Strawn G, Swertz MA, Thompson M, van der Lei J, van Mulligen E, Velterop J, Waagmeester A, Wittenburg P, Wolstencroft K, Zhao J, Mons B (2016). The FAIR guiding principles for scientific data management and stewardship. Sci Data.

[54] Lee PH, Burstyn I (2016). Identification of confounder in epidemiologic data contaminated by measurement error in covariates. BMC Med Res Methodol.

[55] Andrade C (2018). Internal, external, and ecological validity in research design, conduct, and evaluation. Indian J Psychol Med.

[56] Findley MG, Kikuta K, Denly M (2021). External validity. Annu Rev Polit Sci.

[57] Grimes DA, Schulz KF (2002). Bias and causal associations in observational research. Lancet.

[58] Quarto. Available from: https://quarto.org/. Cited 2023 Jun 14.

[59] Dube K, Gallagher T, Gibbons J, MacCaull W (2014). Approach and method for generating realistic synthetic electronic healthcare records for secondary use. Foundations of health information engineering and systems.

[60] Al-Jundi A, Sakka S (2016). Protocol writing in clinical research. J Clin Diagn Res.

[61] OpenAIRE. Argos. Available from: https://argos.openaire.eu/splash/index.html. Cited 2023 May 9.

[62] Papadopoulou E. ARGOS: plan and follow your data. 2021. Available from: https://www.um.edu.mt/library/oar/bitstream/123456789/70269/1/ARGOS_plan_and_follow_your_data_2021.pdf.

[63] Margariti V, Stamati T, Anagnostopoulos D, Nikolaidou M, Papastilianou A (2022). A holistic model for assessing organizational interoperability in public administration. Govern Inform Q.

[64] Weichhart G (2014). Learning for sustainable organisational interoperability. IFAC Proc Vol.

[65] de Mello BH, Rigo SJ, da Costa CA, da Rosa Righi R, Donida B, Bez MR, de Mello BH, Rigo SJ, da Costa CA, da Rosa Righi R, Donida B, Bez MR, Schunke LC (2022). Semantic interoperability in health records standards: a systematic literature review. Health Technol.

[66] Gillespie C, Lovelace R. Efficient R programming: a practical guide to smarter programming. 1st ed. O’Reilly Media, Inc.; 2016. Available from: https://www.oreilly.com/library/view/efficient-r-programming/9781491950777/.

[67] Lutz M. Learning python: powerful object-oriented programming. 5th ed. Sebastopol: O’Reilly Media, Inc.; 2013.

