# Supplementary material for: Federated causal inference based on real-world observational data sources: application to a SARS-CoV-2 vaccine effectiveness assessment
Source: BMC Med Res Methodol. 2023 Oct 23;23:248. doi: 10.1186/s12874-023-02068-3 (PMC10594731; doi:10.1186/s12874-023-02068-3)
Supplement: Supplementary file 2 — Additional file 2. [file 12874_2023_2068_MOESM2_ESM.docx]

**List of participating or contributing members of the BeYond-COVID project**

The described work was conducted within the context of the European BeYond-COVID project, with all authors involved as participants within this project. The authors would like to acknowledge the participation and contribution of additional members of the BeYond-COVID project in the project’s use case.

Participating or contributing members are: Nina Van Goethem^1^, Enrique Bernal-Delgado^3^, Francisco Estupiñán-Romero^3^, Marjan Meurisse^1,2^, Natalia Martínez-Lizaga^3^, Santiago Royo-Sierra^3^, Javier González-Galindo^3^, Simon Saldner^4^, Lorenz Dolanski-Aghamanoukjan^5^, Alexander Degelsegger-Marquez^5^, Stian Soiland-Reyes^6,7^, Vasso Kalaitzi^4^, Claudia Habl^8^, Gunter Maier^9^ (GÖG), Mirjam Knol^10^, Chantal Reusken^10^, Mariken Tijhuis^10^, Leon Schutte^10^, Markus Perola^11^, Teemu Paajanen^11^, Kati Kristiansson^11^, Pekka Jousilahti^11^, Jostein Starrfelt^12^, Hinta Meijerink^12^.

^1^ Department of Epidemiology and Public Health, Sciensano, Brussels, Belgium
^2^ IREC – EPID, Université Catholique de Louvain, Brussels, Belgium
^3^ Data science for Health Services and Policy, Instituto Aragonés de Ciencias de la Salud (IACS), Zaragoza, Spain
^4^ Data Archiving and Networked Services, Royal Netherlands Academy of Arts & Sciences, Amsterdam, The Netherlands
^5^ International Affairs, Policy, Evaluation and Digitalisation, Gesundheit Österreich GmbH (GÖG), Vienna, Austria
^6^ Department of Computer Science, The University of Manchester, Manchester, UK
^7^ Informatics Institute, Universiteit van Amsterdam, Amsterdam, The Netherlands
^8^ International Affairs and Subsidiaries, Gesundheit Österreich GmbH (GÖG), Vienna, Austria
^9^ Planning and system development, Gesundheit Österreich GmbH (GÖG), Vienna, Austria
^10^ National Institute for Public Health and the Environment (RIVM), Bilthoven, The Netherlands
^11^ Finnish Institute for Health and Welfare (THL), Helsinki, Finland
^12^ Norwegian Institute of Public Health (NIPH), Oslo, Norway
